# Drying Effects on Phenolics and Free Radical-Scavenging Capacity of *Rhus pachyrrhachis* and *Rhus virens* Used in Traditional Medicine

**DOI:** 10.3390/molecules24132438

**Published:** 2019-07-02

**Authors:** María Cruz Juárez-Aragón, Yolanda del Rocio Moreno-Ramírez, Antonio Guerra-Pérez, Arturo Mora-Olivo, Fabián Eliseo Olazarán-Santibáñez, Jorge Ariel Torres-Castillo

**Affiliations:** Instituto de Ecología Aplicada, Universidad Autónoma de Tamaulipas, División del Golfo 356. Ciudad Victoria, Tamaulipas 87019, Mexico

**Keywords:** medicinal plants, *Rhus*, phytochemistry, morphoanatomical characterization

## Abstract

*Rhus pachyrrhachis* and *Rhus virens* are medicinal plant species with important uses in northeastern Mexico. They belong to a complex of *Rhus* species called “lantriscos”, which are used for medicinal applications. The medicinal effects of these species are based on traditional use, however, they require phytochemical research to validate their medicinal properties, as well as structural characterization for their correct identification during the collecting practice and uses. The phytochemical potential of aqueous extracts from *R. pachyrrhachis* and *R. virens* was analyzed by the quantification of total phenolic content (TPC), free radical-scavenging potential, and total flavonoids, with a comparison of four drying methods, and some phenolic compounds were identified. Furthermore, the stems and leaves of both species were anatomically characterized to establish a differentiation. *R. pachyrrhachis* and *R. virens* showed similar values of phytochemical contents, although the TPC content (0.17 mg of gallic acid equivalent per gram of dry weight, GAE/g DW) was higher in *R. virens*. The drying method used affected the metabolite contents, and this behavior was related to the species. Regarding the phenolic compounds, shikimic acid, galloylquinic acid, and gallic acid were identified in both species, however, quinic acid was only found in *Rhus pachyrrhachis*, while vanillic acid *O*-hexoside was identified only in *Rhus virens*. At the anatomical level, the pubescence associated with trichomes on the leaves of *Rhus pachyrrhachis* was highlighted as the main differential characteristic.

## 1. Introduction

The use of plants for human health, a recurrent practice in many countries, has influenced the welfare of societies around the world [1]. The beneficial effects of medicinal plants are associated with the presence of secondary metabolites, including phenolic compounds, terpenoids, and alkaloids, among others [2]. Secondary metabolites are considered as the main bioactive elements in traditional medicine [3,4], and some are involved in the elimination of reactive oxygen species (ROS) [5]. Therefore, their importance to human health is related to their antioxidant and free radical-scavenging activities and the prevention of a wide variety of chronic and neurodegenerative diseases [6].

Abdelhalim et al. [7] indicated that the harvest season, environmental conditions in which the plant is developed, processing, and extraction methods are the most influential factors in the yield of active components of medicinal plants. In this sense, studying these factors could ensure the quality of plant materials for consumers [8,9].

One of the main limitations faced by practitioners and users of traditional medicine is related to the correct recognition of morphologically similar species [7], which highlights the need for morphoanatomical and phytochemical characterization of medicinal plants for accurate identification, in order to obtain the benefits of consuming them. The problem originates when some species, due to their similarity, receive the same common name and can be confused when they are marketed, as seen with *Rhus pachyrrhachis* Hemsl. and *Rhus virens* Lindh. Ex A. Gray, both of which are referred to as “lantriscos” in northeast Mexico [10,11].

The genus *Rhus* includes about 200 species, among which are some that have medicinal or nutritional uses [12], although others can cause allergies or poison people [13]. Among the species used in traditional medicine, *R. coriaria* L. has demonstrated anti-inflammatory and protective effects in different neuropathologies in vitro [13], as well as curative effects in complications related to diabetes [14] and therapeutic efficacy in some types of cancer [15]. The analysis and phytochemical characterization of *R. punjabensis* Stewart has allowed researchers to validate its antioxidant, anticancer, and antimicrobial properties [16]. In the same way, *R. leptodictya* Diels has shown important antioxidant and antibacterial activities due to its total phenolic compound and flavonoid content [17]. Also, high phytochemical contents and excellent free radical-scavenging capacities from the fruit and seed oils of *R. chinensis* Mill were seen, with potential for the food and nutritional supplement industries [18,19]. On the other hand, although extracts of some species of this genus are related to cytotoxic effects against transformed cells, they also present toxic effects on normal cells, which highlights the importance of performing integral studies for discovering secondary effects [20,21]. These works showed that the *Rhus* species are sources of phytochemicals, bioactive phenolic compounds, and functional ingredients with multiple potential applications in the pharmaceutical and food industries [22]. However, in the case of the “lantriscos”, used as antidiabetic and antioxidant plants in Mexico, phytochemical studies are lacking and no information about their toxicity is available. Nevertheless, their use as medicinal plants has been noted recently [23,24]. Also, it is unknown whether they have similar composition to justify their use equally, so studies that support their use and consumption are required. 

As mentioned above, the drying method is a determining factor in the phytochemical and antioxidant composition of dried medicinal plants [25]. Under this scheme, the drying of plant materials must be carried out so as to ensure that the phytochemical contents are not altered, together with hygienic, economical management, compatible with traditional practices. However, in the case of traditional practices, sometimes the drying process does not include mechanisms to ensure these aspects and it is necessary to develop strategies to cope with these technical necessities to ensure plant material quality.

Therefore, this study presents total phenolic compound content, free radical-scavenging capacity, and total flavonoids in aqueous extracts of *Rhus pachyrrhachis* and *Rhus virens*, as well as a comparison of drying methods in terms of the phytochemical composition patterns and identification of phenolic compounds. Finally, an anatomical characterization of both species is presented in an attempt to contribute to their correct identification.

## 2. Results

### 2.1. Determination of Phenolic Compounds and Free Radical-Scavenging Capacity

Contents of total phenolic content (TPC) and total flavonoid (TF), and free radical-scavenging capacities on *R. pachyrrhachis* and *R. virens* were evaluated together with drying effects regarding four different drying methods, which included the traditional method (1), carried out by exposing the plant material to shade drying and environmental temperature according to traditional practices; the conventional method (2), where plant material was submitted to drying under shade indoors with temperatures between 28 and 30 °C; drying with a convection oven (3) at 45 °C, and finally, drying inside a prototype (drying method 4), under similar conditions to those used for traditional drying, but in more clean and confined conditions. 

**Figure 1:**
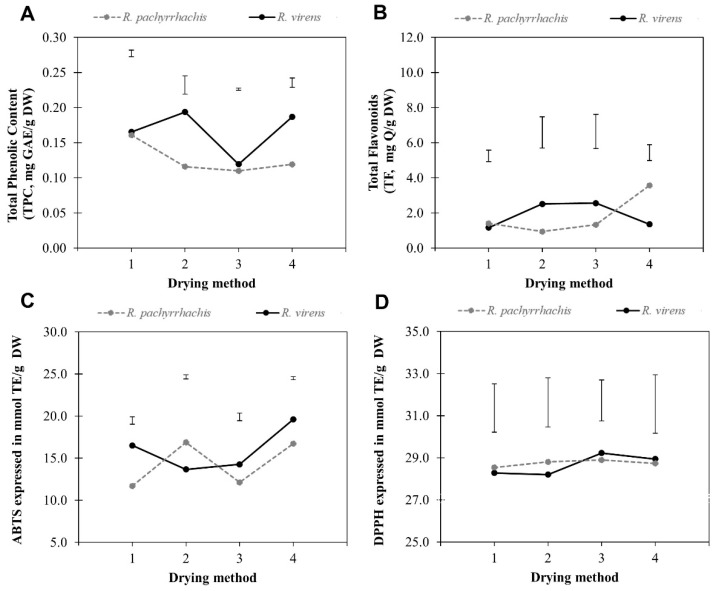
Marginal means of total phenolic content (TPC) (**A**), total flavonoid (TF) (**B**), and free radical-scavenging capacity against 2,2′-azino-bis(3-ethylbenzothiazoline-6-sulfonic acid) (ABTS) (**C**) and 2,2-diphenyl-1-picrylhydrazyl DPPH (**D**) radicals of *Rhus pachyrrhachis* and *Rhus virens*. Vertical bars represent the Tukey’s honest significant difference (HSD, *p* ≤ 0.05) appropriated to compare the species in the same drying method.

The statistical analysis showed the interaction between species and the type of drying method through the four evaluated parameters. The magnitude of the interaction was different for each metabolite (Figure 1). In the case of TPC, *R. pachyrrhachis* presented a trend to lower content in relation to *R. virens* and an interaction was observed with drying methods 1 and 3, but not with drying methods 2 and 4 (Figure 1A), where shade drying could have had an effect on the stability of TPC. In the case of TF content, the interaction showed that both species presented a similar behavior in the drying method 1, but they were different in methods 2 to 4, although in drying methods 2 and 3, *R. pachyrrhachis* had lower values than *R. virens*, while the behavior in method 4 was the opposite (Figure 1B). At species level, *R. pachyrrhachis* and *R. virens* showed variations in the free radical-scavenging activities against 2,2′-azino-bis(3-ethylbenzothiazoline-6-sulfonic acid) (ABTS) (Figure 1C), where *R. pachyrrhachis* presented lower values in drying methods 1, 3, and 4, in contrast to method 2 with higher values than *R. virens*. In the case of the free radical-scavenging against 2,2-diphenyl-1-picrylhydrazyl (DPPH) it was observed that both species showed a very close interaction and a similar behavior with all drying methods (Figure 1D). The interaction drying method × species was present throughout the phytochemical and free radical-scavenging capacities and furthermore, through the differences in contents of all parameters, according to the drying method. 

Considering all drying methods as global aggregated results for the mean comparison indicated that their TPC contents were statistically different, but no differences were registered in the case of the remaining analyzed parameters (Table 1). In general, *R. virens* was superior in TPC and TF contents and free radical-scavenging capacities when compared to *R. pachyrrhachis*, although the statistical test only showed that the most significant difference was identified in TPC with approximately 0.04 mg of gallic acid equivalent per gram of dry weight (GAE/g DW) difference between both species (Table 1). In testing for radical DPPH and TF, both species showed highly similar values, similar to the free radical-scavenging capacity against ABTS. In the latter, the values were 14.35 and 16.01 mmol of Trolox equivalent (mmol TE/g DW), with a difference of 1.66 mmol TE/g DW, and although the statistical test did not indicate dissimilarity between species, it seemed to be apparently different. In general, *R. virens* surpassed *R. pachyrrhachis* in TPC and free radical-scavenging capacity against ABTS and TF. 

### 2.2. Drying Methods

The comparison of drying method effects showed a differential variation for TPC, free radical-scavenging capacity, and TF content between the two species (Table 2). A comparison of means showed significant differences between drying methods as follows: The highest yield of TPC for *R. pachyrrhachis* was observed when drying method 1 was used, and for *R. virens* when drying methods 2 and 4 were used. The highest TF content was detected in *R. pachyrrhachis* with drying method 4. In the case of free radical scavenging capacity against ABTS, for *R. pachyrrhachis*, the highest contents were observed in drying methods 2 and 4, method 4 showed the highest ABTS level for *R. virens*. Drying methods 3 and 4 showed the highest free radical-scavenging capacity against DPPH levels for *R. virens*. No statistical differences were observed in DPPH scavenging capacity determinations for *R. pachyrrhachis*, and no differences were detected for TF contents in *R. virens*. The TPC content observed in both species seemed to be lower with drying method 3, corresponding to the convection oven, with a higher constant temperature and shorter drying time. Important variability was observed in all determinations in that no behavior corresponded to a specific drying method, which reiterates the differential effect attributed to the interaction of drying method and species, which must be considered as part of the processing prior to use. 

### 2.3. Identification of Phenolic Compounds

Of the nine phenolic compounds that were detected using ultra-performance liquid chromatography (UPLC) separation coupled to mass spectrometry analysis (Table 3), a tentative identity assignment of seven was carried out, according to the fragmentation pattern: quinic acid, (−)-shikimic acid, galloyl-hexoside, gallic acid, galloylquinic acid, protocatechuic acid, and vanillic acid *O*-hexoside. Fragmentation patterns of two of the compounds did not allow any association with known phytochemicals and were designated as unknown. Based on these results, differences were observed in the phenolic patterns of *R. pachyrrhachis* and *R. virens*. The presence of (−)-shikimic acid, galloyl-hexoside, gallic acid, and galloylquinic acid was common in both species, and they shared 45% similarity in their phenolic profiles. The 55% dissimilarity in phenolic composition was determined by the exclusive presence of quinic acid and protocatechuic acid for *Rhus pachyrrhachis*, along with two unknown compounds (3.518 and 4.973 Rt). On the other hand, the presence of vanillic acid *O*-hexoside and one unknown compound (3.451 Rt) was observed in *R. virens*, which indicates that *Rhus pachyrrhachis* presented a greater diversity of phenolic compounds. Most of the phenolic compounds detected were phenolic acids. 

### 2.4. Anatomical Description

**Figure 2:**
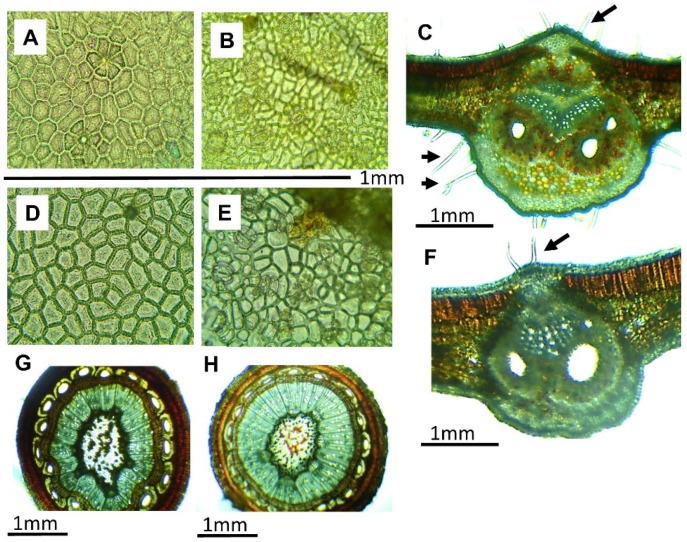
Anatomical description of leaflets and stems of *Rhus pachyrrhachis* and *Rhus virens*: (**A**) adaxial epidermis of *R. pachyrrhachis* observed at 40×; (**B**) abaxial epidermis of *R. pachyrrhachis* at 10×; (**C**) cross-section of *R. pachyrrhachis* leaflet at 10×; (**D**) adaxial epidermis of *R. virens* observed at 40×; (**E**) abaxial epidermis of *R. virens* at 40×; (**F**) cross-section of *R. virens* leaflet at 10×; (**G**) cross-section of *R. pachyrrhachis* stem at 10×, and (**H**) cross-section of *R. virens* stem at 10×. Black arrows indicate positions of trichomes.

The histological analysis of both species showed some useful characteristics to differentiate between the species during the collection process (Figure 2). *Rhus* species presents compound leaves, and every segment of the foliar surface is considered as a unit, called a leaflet. The leaflets of *R. pachyrrhachis* presented an adaxial epidermis (Figure 2A), and cells with thin primary walls, very pubescent (slightly hairy to the touch), with simple unicellular trichomes measuring 0.9 mm in length (such long epidermal cells are responsible for pubescence), an average of 10 trichomes per mm^2^, and without stomata. The abaxial surface (Figure 2B) presented cells with primary walls, pubescent, with trichomes up to 0.7 mm in length, and anomocytic stomata (stomata surrounded by subsidiary cells similar to the rest of the epidermal cells), with a density of 12 stomata per mm^2^. The adaxial epidermis of *R. virens* (Figure 2C) was similar to that of *R. pachyrrhachis*, with an average of 5 trichomes per mm^2^, 0.22 mm on average, with no stomata. The abaxial surface of *R. virens* (Figure 2D) did not present trichomes, and the stomata were anomocytic with a density of 20 stomata per mm^2^. In cross-sections of mature leaflets, a similar histological arrangement was observed for both species (Figure 2E,F), and from the adaxial to the abaxial direction the tissue distribution started with an unstratified adaxial epidermis, followed by two rows of palisade chlorenchyma, under which an accumulation of spongy chlorenchyma with three to six rows was observed. The main vascular bundle showed primary phloem in the central part, and a primary xylem accumulation was observed toward the central lower part. The central vascular bundle was protected by lacunar collenchyma on both sides. The stem analyzed in the cross-section showed the beginnings of secondary growth, although it was taken from tender parts. It presented an epidermis, followed inward by an accumulation of primary cells with thickened walls, similar to the basal organization of cork. In the cortical zone, parenchymal cells appeared, some with orange to brown contents. In addition, some ducts of regular distribution with ovoid openings surrounded by fibers were observed. In the vascular zone, a continuous distribution of the vascular cylinder was observed, with phloem in the most external part surrounding the xylem. In the innermost part of the stem, a parenchymatous medulla with cells with wide lumens and primary walls was found. Some of the cells had clear lumens, while others presented lumens with orange–light brown contents whose composition was unknown. The most noticeable difference between the species was the pubescence in leaves with respect to the quantity and length of the trichomes and, especially, pubescence in the abaxial epidermis of *R. pachyrrhachis*. 

## 3. Discussion

TPC and TF, together with free radical-scavenging capacity, are directly related to the antidiabetic properties of some plants [4]. In fact, antioxidant- and phenolic-enriched products have been used to complement antidiabetic therapies [34,35,36], this supports the idea that *R. pachyrrhachis* and *R. virens* could be used as traditional agents for such a purpose given their TPC and TF contents and free radical-scavenging capacity, although other studies must be undertaken to confirm whether they have antidiabetic effects. The use of aqueous infusions is a common and recommended form of ingesting these species, which means that the existence of phenolic compounds in these preparations confirms their medicinal potential.

Although both species are closely related and collected in a common climatic niche, their general phenolic compound contents and free radical-scavenging capacities were different. This coincides with the different accumulation patterns of phytochemicals observed between closely related species and even in plants of the same variety, indicating that accumulation patterns are influenced by environmental and endogenous parameters [37,38,39]. In the case of the *Rhus* genus, it has been reported that phenolic compounds and antioxidants vary between species and between populations of the same species. Previously, it was demonstrated that *Rhus pentaphylla* Desfontaines and *Rhus tripartita* (Ucria) Grande had interspecific and interpopulation variations of both species, and differential accumulation patterns in the stems, leaves, and fruits of each population were observed [40], which was probably influenced by growth conditions and must be considered when developing strategies for the use and management of *Rhus* species.

The TPC and TF contents observed in *R. pachyrrhachis* and *R. virens* were lower than those observed in samples of several populations of *R. pentaphylla*, *R. tripartita*, and *R. leptodictya*, which were extracted with organic solvents [17,40], however, values of *R*. *pachyrrhachis* and *R. virens* were superior to the values reported for *Rhus punjabensis* extracted using 11 solvents [14]. In an additional comparison, TF contents were higher than those observed in leaves of *R. punjabensis* [14], but were lower than those reported for *R. leptodictya* [17]. This indicates that the contents in the species studied here are in the range of that of other studies even when different solvents were used, probably due to a high abundance of these kinds of compounds. In this respect, the relevant influence of solvents for such extractions must be highlighted, and this needs to be considered for industrial or medicinal applications. The free radical scavenging capacity against both radicals was higher than the values reported for *Rhus succedanea* L. [41], however, free radical-scavenging capacity against ABTS was lower than that reported for *R. chinensis* obtained with water and methanol [42]. This highlights the wide variation in free radical-scavenging levels in representatives of the *Rhus* species, pointing out the importance of solvent and extraction procedures. In this work, extraction was performed with water in order to create conditions similar to those in the common uses of these species, and results confirmed the presence of phenolic compounds and free radical-scavenging capacity, which is thought to occur in traditional preparations. Also, levels of water extraction used in this work, in general, presented content levels similar to other studied species of the *Rhus* genus.

Regarding the contents of phenolic compounds, antiradical activities should be considered during the processing, particularly in drying. It has been reported that the various drying procedures affect the quality and quantity of metabolites present in plant matter [43,44], so studies are needed on the choice of the best method. In this work, different drying methods were evaluated that are relatively accessible to collectors and practitioners of traditional medicine in rural areas of Mexico. Drying in the shade is the most established traditional practice for the ancestral uses of medicinal plants [45], however, this implies management practices that expose plant materials to slow drying and contact with physical (dust, fibers, garbage) and biological (fungi, rodents, insects) contaminants, which should be avoided to ensure the quality and safety of materials. Due to this, we evaluated the variation of phytochemicals in dried material in a prototype, made with metal mesh, that recreated drying in the shade but in a confined way. When compared with other drying variants, it was observed that the effects were similar to the traditional method and conventional drying, whereas using the forced-air oven affected the contents, probably due to higher temperatures. The interaction between the drying method and species showed a significant difference, and while the species responded similarly to each drying method, it should be noted that *R. virens* showed greater variability, which could be related to different effects on the analyzed metabolite and the plant species.

The identification of phenolic compounds and the detected activities in this work coincide, in most cases, with those reported for other *Rhus* species [17,46,47], many of which are related to medicinal or nutritional uses. Among the main components of phenolic nature, phenolic acids and some of their derivatives stand out. Gallic acid has been indicated as the main bioactive agent of some species of the genus, and for the fruits of *R. coriaria* it has been identified as the main phenolic acid and the most active antioxidant, although protocatechuic acid and vanillic acid are also present. In addition, the presence of cyanidin glycosides, peonidin, pelargonidin, petunidin, and delphinidin is recognized [48,49,50]. In *Rhus verniciflua* Stokes, gallic acid was the main active component from the bark of the stem, and has been reported to have cytotoxic activity against HeLa cells [51]. It has also been reported that an ethanolic extract of the vegetative part of *R. verniciflua* reduced cell death in a macrophage model, and the extract contained *p*-coumaric acid, fustin, kaempferol-3-*O*-glucoside, sulfuretin, and butein [52]. From a bark extract of *R. verniciflua*, positive effects were obtained against oxidative stress in human cell lines. That extract presented a composition with predominantly gallic acid, 2-(ethoxymethoxy)-3-hydroxyphenol, fustin, a fustin isomer, tetragalloyl glucose, pentagalloyl glucose, fisetin, sulfuretin, a sulfuretin isomer, butein, and three unidentified compounds [53]. Finally, from the fruit of *R. coriaria*, 221 compounds were identified, of which 180 were reported for the first time for this fruit, and some of them coincided with those detected in the extracts of the species studied in this work. This highlights the importance of exploring these species to consider them as potential sources of various bioactive compounds [2]. These arguments support the presence of phenolic acids and their derivatives in the leaves of *R. pachyrrhachis* and *R. virens* and also their medicinal use.

The anatomical descriptions of both species allow us to recognize that the most evident differences between them are at the epidermal level, since the histological arrangement is very similar between the mature leaves and stems analyzed; the pubescence of the leaves was the most evident characteristic, whereas the density and length of trichomes from the adaxial epidermis of *R. pachyrrhachis* allowed their differentiation, which can be done by rubbing the leaves, so touching the leaves during collection could allow differentiation, at least between these species. On the other hand, the density of stomata in the abaxial epidermis was also a differential characteristic, where *R. virens* showed the greater amount. Differentiating and characterizing these species is necessary to ascertain the factors that could influence the medicinal effects associated with the phytochemical contents and homogeneity of the materials. This should be considered to ensure the quality of preparations and products derived from medicinal plants [54,55]. Both species coexist in the study area and may even be growing together, which makes collectors and practitioners of traditional medicine manage them collectively. Therefore, differentiation with respect to pubescence and detected compounds is a starting point to guide collectors to ensure the homogeneity of raw materials, and thus ensure that the beneficial effects can be specifically associated with a known material.

## 4. Materials and Methods

### 4.1. Collection Site, Plant Material, and Extract Preparation

The collection site of natural populations of *R. pachyrrhachis* and *R. virens* was defined from the previous taxonomic identification of both species with herbarium specimens (UAT 02,848 and UAT 02629 respectively), and was located in Jaumave, Tamaulipas, Mexico (for *R. pachyrrhachis*: 23°37′10.87″ N, 99°16′7.32″ W, and for *R. virens*: 23°37′3710″ N; 99°12′3586″ W). Sample collection was performed near rural roads, where traditional gatherers collect plants.

The plant material was washed with running water and then divided into 4 subsamples that were submitted to different drying methods: (1) Traditional drying (traditional), which is used by collectors, consisted of placing the plant material over a clean blanket on the floor to dry the plants in the open air and in the shade at temperatures between 25 and 37 °C for 6 days, the temperature variation was due to the natural environmental conditions. (2) Drying in the shade indoors (conventional) with temperatures between 28 and 30 °C for 6 days. (3) Drying in a convection oven (oven) at 45 °C for 3 days. (4) Drying in the shade inside a drying device (prototype) at a temperature range of 25 to 37 °C for 6 days (similar to traditional drying, but in more clean and confined conditions). This device is a prototype for hygienic drying made with metal mesh to facilitate aeration, which has been proposed for use in rural areas. The prototype has the drying compartment 50 cm above floor level, which allows aeration in all directions.

For extraction, 1 g of powdered plant material was mixed by stirring with distilled water at a ratio of 1:4 (*w*/*v*) for 20 min, followed by centrifugation at 10,000× *g* for 10 min. The clarified extract of each sample was recovered from the supernatant and stored at −20 °C until use in subsequent analysis. Total volume of each extraction was registered for subsequent calculations. Each extraction was done in triplicate. 

### 4.2. Total Phenolic Content (TPC)

TPC was determined using aliquots of each extraction. Total volume per gram was registered. TPC was quantified with Folin–Ciocalteu reagent [56], the reactions used 5 μL of extract and 245 μL of water, mixed with 125 μL of 1 N Folin–Ciocalteu reagent (Sigma-Aldrich, St. Louis, MO, USA), followed by incubation in the dark for 5 min. Subsequently, 625 μL of 20% Na_2_HCO_3_ (CTR, Monterrey, Nuevo Leon, Mexico) was added to each reaction. The reactions were incubated for 2 h in the dark, and then their absorbance at 760 nm was recorded with a UV–Vis spectrophotometer (UV-6000, Metash Instruments Co. Ltd., Shanghai, China). For the quantification of total phenolics, gallic acid (3,4,5-trihydroxybenzoic acid) (Sigma-Aldrich, St. Louis, MO, USA) was used in concentrations of 0.001 to 0.008 mg/mL in a standard curve. The standard curve was prepared in the same conditions of extract reactions. The TPC of each sample was determined according to the volume used in each reaction, and then extrapolated to the total volume extracted per gram of dry weight material. TPC was expressed in milligram equivalents of gallic acid per gram of dry weight. Each reaction was done in triplicate.

### 4.3. Total Flavonoid (TF)

The total flavonoid content was quantified with the colorimetric method of aluminum chloride [57]. To obtain the standard curve for flavonoid quantification expressed as quercetin equivalent, solutions were prepared in concentrations of 0.1 to 0.5 mg/mL. Subsequently, 100 μL of standard or sample solution was mixed with 300 μL of 95% ethanol, 20 μL of 10% aluminum chloride, 20 μL of 1 M potassium acetate, and 560 μL of distilled water, shaken, and incubated for 30 min at a temperature of 28–30 °C. Absorbance of the reactions was recorded at 415 nm. The TF content was determined for each reaction and then related to the total volume obtained from 1 g of dry weight material. All flavonoids were expressed in terms of milligrams of quercetin (mg Q)/g DW. Each reaction was done in triplicate.

### 4.4. Free Radical-Scavenging Activity against 2,2-diphenyl-1-picrylhydrazyl (DPPH)

Antiradical capacity was analyzed with an assay using DPPH, as proposed by Brand-Williams et al. [58]. A solution of DPPH (600 μM) (Sigma-Aldrich, St. Louis, MO, USA) in absolute methanol (CTR, Monterrey, Nuevo Leon, Mexico) was prepared, with absorbance adjusted to 0.700 nm with the UV–Vis spectrophotometer at 515 nm. The standard curve was prepared with Trolox^®^ (Sigma-Aldrich, St. Louis, MO, USA) with concentrations of 0.1, 0.2, 0.4, 0.6, 0.8, 1, and 1.2 mM. Subsequently, 975 μL of the DPPH solution was mixed with 25 μL of standard or extract solution, and incubated in the dark for 30 min. The free radical-scavenging capacity was determined based on the change in absorbance at 515 nm, according to the standard curve. The free radical scavenging capacity of each sample was determined according to the volume used in each reaction and then extrapolated to the total volume extracted per gram of dry weight material. Finally, it was expressed in mmol TE/g DW. Each reaction was done in triplicate.

### 4.5. Free Radical Scavenging Activity against 2,2′-azino-bis(3-ethylbenzothiazoline-6-sulfonic Acid (ABTS)

The free radical scavenging capacity against the ABTS radical was measured according to Re et al. [59]: 10 μL of sample or standard solution was mixed with 1 mL of working solution of the ABTS radical. The stock solution was obtained by mixing 7 mM ABTS and 2.45 mM potassium persulfate (Sigma-Aldrich, St. Louis, MO, USA) in distilled water, and this mixture was incubated for 16 h at 25 ± 2 °C in the dark prior to use, then the absorbance of this solution was adjusted to 0.7 at 732 nm using absolute ethanol (CTR, Monterrey, Nuevo León, Mexico). The absorbance of the reaction mixtures was recorded after 6 min of reaction and compared with a calibration curve of Trolox^®^ prepared with concentrations of 0.1, 0.2, 0.4, 0.6, 0.8, 1, and 1.2 mM. The concentration was expressed in mmol TE/g DW, as indicated above for DPPH calculations. Each reaction was done in triplicate. 

### 4.6. Identification of Phenolic Compounds by Ultra-Performance Liquid Chromatography-Electrospray Ionization Coupled to Mass Spectrometry Elevated Energy with Quadrupole-Time of Flight Analyzer (UPLC-ESI-Q/TOF-MSe)

Aqueous extracts of recently shaded dried leaves were fractionated with a stationary phase (Amberlite XAD-16N) and a mobile phase (70% ethanol) to recover enriched fractions of the compounds of interest. Chromatographic separation of the phenolic compounds was carried out on an Acquity ultra-performance liquid chromatography (UPLC) system, connected to an auto-sampler and a binary pump with a 10 μL loop. Chromatographic separations were performed according to Kumari et al. [60], with slight modifications, using a BEH Phenyl Column (2.1 mm × 100 mm, 1.7 μm; Waters, Elstree, Herts, UK) at 40 °C, with a gradient elution and constant flow of 0.3 mL/min. The mobile phase was composed of phase A, water acidified with formic acid at 0.1% (*v*/*v*), and phase B, acetonitrile (100%). The gradient used was 97% of phase A for 1 min, before the proportion of phase B was changed from 5% to 15% from minute 1 to minute 4.40. After this, 15% of B was used for 4.60 min before being returned to initial conditions for 1 min. The UPLC system was coupled to a mass quadrupole-time of flight (Q-TOF) orthogonal accelerated spectrometer (Q-TOF™, Waters, UK) equipped with an electrospray ionization source. Detection of the mass spectrum was completed in 10 min in the negative ion mode, with a mass range of 50–1200 *m/z*. The optimal values for the source parameters were as follows: capillary voltage 3.0 kV, dry gas temperature 210 °C, gas flow 8.0 L/min, nebulizer pressure 2 bar, and spectrum velocity 1 Hz. Automated tandem mass spectrometry (MS2) assays were done using an energy of slope collision of 15–35 V with argon as the collision gas and adjustment of the exploration time every 1 s. The phenolic compounds in the extracts were identified by their full mass spectra and unique mass fragmentation patterns. Identification of the compounds was primarily conducted by comparing the observed MSe spectra with those found in the literature and the MassBank (http://www.massbank.jp/), ChemSpider (http://www.chemspider.com), and PubChem (https://pubchem.ncbi.nlm.nih.gov) databases.

### 4.7. Anatomical Description

Descriptions of leaflets and stems were conducted using handmade sections. The samples were fixed in a solution of formaldehyde, absolute ethanol, acetic acid, and distilled water (10:20:20:50 (*v*/*v*)) for 12 h. Subsequently, the samples were rinsed with distilled water and cross-sections of each structure were made for microscopic observation. The natural pigmentation of the tissues allowed for description without staining the fixed tissue, only 0.1% safranin was used to confirm the presence of lignin in the sclerenchyma and xylem. For the epidermis samples, a count of stomata and trichomes per mm^2^ was carried out with the objective of 10× in three fields to establish a density range. The epidermis was obtained by scraping the mesophyll from the leaflet with a razor, on the side opposite to the desired epidermis for observation.

### 4.8. Statistical Analysis

Field samples of leaves and stems were obtained from a total of 20 individuals randomly sampled from each species. Each phytochemical determination was performed three times. The values obtained were analyzed through a factor analysis, marginal means and a means comparison applying the Tukey test (*p* < 0.05) with the SAS v. 9.3 statistical package [61].

## 5. Conclusions

*R. pachyrrhachis* and *R. virens* showed phenolic compounds and free radical-scavenging capacity at similar levels compared to other *Rhus* species. TPC and free radical-scavenging capacity against ABTS were the phytochemical characteristics that distinguished these two species. The determination of DPPH radical and TF were similar. The drying method used affected both species and their scavenging capacity. Based on the determination of TPC, TF, and free radical-scavenging capacity, the prototype drying method was the best for both species. Each species presented a particular phenolic composition. Among the differences at the anatomical level, pubescence was highlighted as a relevant characteristic during handling by the collectors.

## Figures and Tables

**Table 1:** Global aggregated results of TPC, TF, and free radical-scavenging capacity against ABTS and DPPH radicals of *Rhus pachyrrhachis* and *Rhus virens*.

Species	Free Radical-Scavenging Capacity			
	TPC	TF	ABTS	DPPH
*Rhus pachyrrhachis*	0.13 ± 0.02 ^b^	1.81 ± 1.21 ^a^	14.35 ± 2.79 ^a^	28.74 ± 0.18 ^a^
*Rhus virens*	0.17 ± 0.03 ^a^	1.85 ± 1.03 ^a^	16.01 ± 2.53 ^a^	28.66 ± 0.47 ^a^
HSD	0.006	0.54	0.95	0.13

TPC expressed in mg gallic acid equivalent per gram of dry weight (GAE/g DW), total flavonoids (TF) expressed in mg of quercetin (Q)/g DW, and values of DPPH and ABTS expressed in mmol TE/g DW. Different letters in the same column indicate significant differences (Tukey’s honest significant difference (HSD)).

**Table 2:** Comparison of means between four drying methods for total phenolic content (TPC, mg GAE/g of DW), contents of total flavonoid (TF, mg Q/g DW), and free radical-scavenging capacity against DPPH (mmol TE/g DW) and ABTS (mmol TE/g DW) quantified in leaf extracts of *Rhus pachyrrhachis* and *Rhus virens*.

Drying Method	*Rhus pachyrrhachis*				*Rhus virens*			
	TPC	TF	ABTS	DPPH	TPC	TF	ABTS	DPPH
1. Traditional	0.16 ^A^	1.41 ^B^	11.70 ^B^	28.54 ^A^	0.17 ^b^	1.17 ^a^	16.50 ^b^	28.28 ^b^
2. Conventional	0.12 ^B^	0.94 ^B^	16.88 ^A^	28.81 ^A^	0.19 ^a^	2.51 ^a^	13.66 ^c^	28.20 ^b^
3. Oven	0.11 ^B^	1.32 ^B^	12.13 ^B^	28.90 ^A^	0.12 ^c^	2.56 ^a^	14.27 ^c^	29.23 ^a^
4. Prototype	0.12 ^B^	3.57 ^A^	16.71 ^A^	28.73 ^A^	0.18 ^a^	1.35 ^a^	19.61 ^a^	28.94 ^a^
HSD	0.021	1.693	3.456	0.373	0.013	1.618	2.154	0.433

HSD, honest significant difference. Different letters in the same column show significant differences (Tukey test, *p* < 0.05).

**Table 3:** Compounds from *Rhus pachyrrhachis* and *Rhus virens* identified by UPLC–Mse (Ultra performance liquid chromatography–mass spectrometry elevated energy).

Peak	Rt (min)	[M − H] (*m/z*)	MS2 Dominant Fragment Ion	Tentative Assignment	Molecular Formula	*R. pachyrrhachis*	*R.* *virens*	References
1	0.846	191.0871	-	Quinic acid	C_7_H_12_O_6_	*		[26,27,28]
2	0.913	173.0794	93.0801, 173.08, 155.07, 137.06, 111.08, 93.07	(−)-Shikimic acid	C_7_H_10_O_5_	*	*	[29]
3	1.353, 1.319	331.0622, 331.0648	278.98, 232.98, 242.97, 212.05, 200.91, 191.03, 211.05, 174.98, 169.05, 169.04, 151.04, 125.06	Galloyl-hexoside	C_13_H_16_O_10_	*	*	[30]
4	1.522, 1.556	125.0672, 125.0655	169.05, 151.96, 113.02, 101.98, 97.07	Gallic acid	C_7_H_6_O_5_	*	*	[31]
5	1.725, 1.692	191.0862, 191.0880	365.03, 343.05, 169.05, 167.03, 153.06, 123.05	Galloylquinic acid	C_14_H_16_O_10_	*	*	[30]
6	2.571	109.0727	153.05, 108.06	Protocatechuic acid	C_7_H_6_O_4_	*		[32]
7	3.518, 3.451	123.0869, 123.0879	331.10, 285.11, 285.11, 181.00, 174.99, 161.08, 143.07, 131.00, 113.02	UK	-	*	*	-
8	4.973	359.1374 (100)	341.13, 187.10, 160.09	UK	-	*		-
9	5.751	167.0714 (100)	191.06, 123.08	Vanillic acid *O*-hexoside	C_14_H_18_O_9_		*	[33]

UK, unknown compound; * indicates presence.

## References

[1] Teixidor-Toneu I., Jordan F.M., Hawkins J.A. (2018). Comparative phylogenetic methods and the cultural evolution of medicinal plant use. Nat. Plants.

[2] Shakya A.K. (2016). Medicinal plants: Future source of new drugs. Int. J. Herb. Med..

[3] Wink M. (2015). Modes of Action of Herbal Medicines and Plant Secondary Metabolites. Medicines.

[4] Ota A., Ulrih N.P. (2017). An Overview of Herbal Products and Secondary Metabolites Used for Management of Type Two Diabetes. Front. Pharmacol..

[5] Fu X., Guo H., Cong W., Du H., Meng X. (2017). Herbal medicine Radix Scutellariae quality improved by exposure of the fresh root to high temperature. Orient. Pharm. Exp. Med..

[6] Losada-Barreiro S., Bravo-Díaz C. (2017). Free radicals and polyphenols: The redox chemistry of neurodegenerative diseases. Eur. J. Med. Chem..

[7] Abdelhalim A., Aburjai T., Hanrahan J., Abdel-Halim H. (2017). Medicinal Plants Used by Traditional Healers in Jordan, the Tafila Region. Pharmacogn. Mag..

[8] Canter P.H., Thomas H., Ernst E. (2005). Bringing medicinal plants into cultivation: opportunities and challenges for biotechnology. Trends Biotechnol..

[9] Ekor M. (2014). The growing use of herbal medicines: issues relating to adverse reactions and challenges in monitoring safety. Front. Pharmacol..

[10] González-Rojas J.I., Farquhar C.C., Guerrero-Madriles M., Ballesteros-Medrano O., Núñez-Gonzalí A. (2014). Breeding Records of Black-capped Vireo (*Vireo atricapilla* ) in Northeastern Mexico. Wilson J. Ornithol..

[11] Estrada-Castillo E., Villarreal-Quintanilla J.Á., Rodríguez-Salinas M.M., Encinas-Domínguez J.A., González-Rodríguez H., Figueroa G.R., Arévalo J.R. (2018). Ethnobotanical Survey of Useful Species in Bustamante, Nuevo León, México. Hum. Ecol..

[12] Morshedloo M.R., Maggi F., Neko H.T., Aghdam M.S. (2018). Sumac (*Rhus coriaria* L.) fruit: Essential oil variability in Iranian populations. Ind. Crop. Prod..

[13] Barkley F.A. (1934). Poison Ivy and Poison Sumac as an Etiologic Factor in Contact Dermatitis in the Central States. Proc. Okla. Acad. Sci..

[14] Tabassum S., Ahmed M., Mirza B., Naeem M., Zia M., Shanwari Z.K., Khan G.M. (2017). Appraisal of phytochemical and in vitro biological attributes of an unexplored folklore: Rhus Punjabensis Stewart. BMC Complement. Altern. Med..

[15] Doğan A., Çelik I. (2016). Healing effects of sumac (*Rhus coriaria*) in streptozotocin-induced diabetic rats. Pharm. Boil..

[16] Muazzam A., Dalrymple M.B., Whetton A.D., Townsend P.A. (2018). Can Rhus Coriaria be a Potential, Natural, Treatment for Prostate Cancer?. Canc. Sci. Onchol..

[17] Mtunzi F.M., Ejidike I.P., Matamela T., Dikio E., Klink M.J. (2017). Phytochemical Profiling, Antioxidant and Antibacterial Activities of Leaf Extracts From Rhus leptodictya. Int. J. Pharmacogn. Phytochem. Res..

[18] Zhang C., Ma Y., Gao F., Zhao Y., Cai S., Pang M. (2018). The free, esterified, and insoluble-bound phenolic profiles of Rhus chinensis Mill. fruits and their pancreatic lipase inhibitory activities with molecular docking analysis. J. Funct. Foods.

[19] Shi L., Zheng L., Liu R., Chang M., Huang J., Zhao C., Jin Q., Wang X. (2019). Potential underutilized oil resources from the fruit and seed of Rhus chinensis Mill. Ind. Crop. Prod..

[20] Son Y.-O., Lee K.-Y., Lee J.-C., Jang H.-S., Kim J.-G., Jeon Y.-M., Jang Y.-S. (2005). Selective antiproliferative and apoptotic effects of flavonoids purified from Rhus verniciflua Stokes on normal versus transformed hepatic cell lines. Toxicol. Lett..

[21] Mirian M., Behrooeian M., Ghanadian M., Dana N., Sadeghi-Aliabadi H. (2015). Cytotoxicity and antiangiogenic effects of Rhus coriaria, Pistacia vera and Pistacia khinjuk oleoresin methanol extracts. Res. Pharm. Sci..

[22] Abu-Reida I.M., Jamous R.M., Ali-Shtayeh M.S. (2014). Phytochemistry, Pharmacological Properties and Industrial Applications of Rhus Coriaria L. (Sumac). Jordan J. Boil. Sci..

[23] Alonso-Castro A.J., Domínguez F., Zapata-Morales J.R., Carranza-Álvarez C. (2015). Plants used in the traditional medicine of Mesoamerica (Mexico and Central America) and the Caribbean for the treatment of obesity. J. Ethnopharmacol..

[24] Maiti R., Rodriguez H.G., Kumari C.A., Sarkar N.C. (2016). Macro and micro-nutrient contents of 18 medicinal plants used traditionally to alleviate diabetes in Nuevo Leon, northeast of Mexico. Pak. J. Bot..

[25] Dhanani T., Shah S., Gajbhiye N., Kumar S. (2017). Effect of extraction methods on yield, phytochemical constituents and antioxidant activity of Withania somnifera. Arab. J. Chem..

[26] Morales-Soto A., Gómez-Caravaca A.M., García-Salas P., Segura-Carretero A., Fernández-Gutiérrez A. (2013). High-performance liquid chromatography coupled to diode array and electrospray time-of-flight mass spectrometry detectors for a comprehensive characterization of phenolic and other polar compounds in three pepper (*Capsicum annuum* L.) samples. Food Res. Int..

[27] Gómez-Romero M., Carretero A.S., Fernández-Gutiérrez A. (2010). Metabolite profiling and quantification of phenolic compounds in methanol extracts of tomato fruit. Phytochem..

[28] Iswaldi I., Gómez-Caravaca A.M., Lozano-Sánchez J., Arráez-Román D., Carretero A.S., Fernández-Gutiérrez A. (2013). Profiling of phenolic and other polar compounds in zucchini (*Cucurbita pepo* L.) by reverse-phase high-performance liquid chromatography coupled to quadrupole time-of-flight mass spectrometry. Food Res. Int..

[29] National Center for Biotechnology Information PubChem Compound Database. https://pubchem.ncbi.nlm.nih.gov/.

[30] Abu-Reidah I.M., Ali-Shtayeh M.S., Jamous R.M., Arráez-Román D., Segura-Carretero A. (2015). HPLC–DAD–ESI-MS/MS screening of bioactive components from *Rhus coriaria* L. (Sumac) fruits. Food Chem..

[31] López-Gutiérrez N., Romero-González R., Vidal J.L.M., Frenich A.G. (2016). Determination of polyphenols in grape-based nutraceutical products using high resolution mass spectrometry. LWT.

[32] Gomez-Romero M., Zurek G., Schneider B., Baessmann C., Segura-Carretero A., Fernández-Gutiérrez A. (2011). Automated identification of phenolics in plant-derived foods by using library search approach. Food Chem..

[33] Karar M.E., Kuhnert N. (2015). UPLC-ESI-Q-TOF-MS/MS characterization of phenolics from Crataegus monogyna and Crataegus laevigata (Hawthorn) leaves, fruits and their herbal derived drops (Crataegutt Tropfen). J. Chem. Biol. Ther..

[34] Martineau L.C., Couture A., Spoor D., Benhaddou-Andaloussi A., Harris C., Meddah B., LeDuc C., Burt A., Vuong T., Le P.M. (2006). Anti-diabetic properties of the Canadian lowbush blueberry Vaccinium angustifolium Ait. Phytomedicine.

[35] Dembinska-Kiec A., Mykkänen O., Kieć-Wilk B., Mykkänen H. (2008). Antioxidant phytochemicals against type 2 diabetes. Br. J. Nutr..

[36] Oyedemi S.O., Yakubu M.T., Afolayan A.J. (2011). Antidiabetic activities of aqueous leaves extract of Leonotis leonurus in streptozotocin induced diabetic rats. J. Med. Plant Res..

[37] Moyer R.A., Hummer K.E., Finn C.E., Frei B., Wrolstad R.E. (2002). Anthocyanins, Phenolics, and Antioxidant Capacity in Diverse Small Fruits: *Vaccinium*, *Rubus*, and *Ribes*. J. Agric. Food Chem..

[38] Nickavar B., Alinaghi A., Kamalinejad M. (2010). Evaluation of the antioxidant properties of five Mentha species. Iran. J. Pharm. Res..

[39] Chew Y., Lim Y. (2018). Evaluation and Comparison of Antioxidant Activity of Leaves, Pericarps and Pulps of Three Garcinia Species in Malaysia. Free Radic. Biol. Med..

[40] Itidel C., Chokri M., Mohamed B., Yosr Z. (2013). Antioxidant activity, total phenolic and flavonoid content variation among Tunisian natural populations of Rhus tripartita (Ucria) Grande and Rhus pentaphylla Desf. Ind. Crop. Prod..

[41] Surveswaran S., Cai Y., Corke H., Sun M. (2007). Systematic evaluation of natural phenolic antioxidants from 133 Indian medicinal plants. Food Chem..

[42] Cai Y., Luo Q., Sun M., Corke H. (2004). Antioxidant activity and phenolic compounds of 112 traditional Chinese medicinal plants associated with anticancer. Life Sci..

[43] Nguyen V.T., Van Vuong Q., Bowyer M.C., Van Altena I.A., Scarlett C.J. (2015). Effects of Different Drying Methods on Bioactive Compound Yield and Antioxidant Capacity of Phyllanthus amarus. Dry. Technol..

[44] Samoticha J., Wojdyło A., Lech K. (2016). The influence of different the drying methods on chemical composition and antioxidant activity in chokeberries. LWT.

[45] Orphanides A., Goulas V., Gekas V. (2016). Drying technologies: vehicle to high-quality herbs. Food Eng. Rev..

[46] Neffati N., Aloui Z., Karoui H., Guizani I., Boussaid M., Zaouali Y. (2017). Phytochemical composition and antioxidant activity of medicinal plants collected from the Tunisian flora. Nat. Prod. Res..

[47] Kim K., Park K.-I. (2019). A Review of Antiplatelet Activity of Traditional Medicinal Herbs on Integrative Medicine Studies. Evidence-Based Complement. Altern. Med..

[48] Ferk F., Chakraborty A., Simic T., Kundi M., Knasmüller S. (2007). Antioxidant and free radical scavenging activities of sumac (Rhus coriaria) and identification of gallic acid as its active principle. BMC Pharmacol..

[49] Kosar M., Bozan B., Temelli F., Baser K., Baser K.H.C. (2007). Antioxidant activity and phenolic composition of sumac (*Rhus coriaria* L.) extracts. Food Chem..

[50] Romeo F.V., Ballistreri G., Fabroni S., Pangallo S., Nicosia M.G.L.D., Schena L., Rapisarda P. (2015). Chemical Characterization of Different Sumac and Pomegranate Extracts Effective against Botrytis cinerea Rots. Molecules.

[51] Kim J.B. (2003). Identification of antioxidative component from stem bark of Rhus verniciflua. J. Korean Food Nutr..

[52] Jung C.H., Jun C.-Y., Lee S., Park C.-H., Cho K., Ko S.-G. (2006). Rhus verniciflua Stokes Extract: Radical Scavenging Activities and Protective Effects on H2O2-Induced Cytotoxicity in Macrophage RAW 264.7 Cell Lines. Boil. Pharm. Bull..

[53] Liu J., Jia L., Kan J., Jin C.-H. (2013). In vitro and in vivo antioxidant activity of ethanolic extract of white button mushroom (*Agaricus bisporus*). Food Chem. Toxicol..

[54] Street R., Stirk W., Van Staden J., Street R. (2008). South African traditional medicinal plant trade—Challenges in regulating quality, safety and efficacy. J. Ethnopharmacol..

[55] Jamshidi-Kia F., Lorigooini Z., Amini-Khoei H. (2018). Medicinal plants: Past history and future perspective. J. Herbmed Pharmacol..

[56] Singleton V.L., Orthofer R., Lamuela-Raventos R.M. (1999). [14] Analysis of total phenols and other oxidation substrates and antioxidants by means of folin-ciocalteu reagent. Methods in Enzymol..

[57] Chang C.C., Yang M.H., Wen H.M., Chern J.C. (2002). Estimation of total flavonoid content in propolis by two complementary colorimetric methods. J. Food Drug Anal..

[58] Brand-Williams W., Cuvelier M., Berset C. (1995). Use of a free radical method to evaluate antioxidant activity. LWT.

[59] Re R., Pellegrini N., Proteggente A., Pannala A., Yang M., Rice-Evans C. (1999). Antioxidant activity applying an improved ABTS radical cation decolorization assay. Free. Radic. Boil. Med..

[60] Kumari S., Elancheran R., Kotoky J., Devi R. (2016). Rapid screening and identification of phenolic antioxidants in Hydrocotyle sibthorpioides Lam. by UPLC–ESI-MS/MS. Food Chem..

[61] SAS Institute (2011). SAS User’s Guide: Statistics, Version 9.3.

